# A model for academic institution support for community-engaged research

**DOI:** 10.1017/cts.2017.295

**Published:** 2017-11-16

**Authors:** Dennis P. Scanlon, Laura J. Wolf, Cynthia H. Chuang, Jennifer L. Kraschnewski, Eugene J. Lengerich, Susan M. McHale, Ian M. Paul, Janice Penrod

**Affiliations:** 1 Department of Health Policy and Administration, Penn State University, State College, University Park, PA, USA; 2 Center for Health Care and Policy Research, Penn State University, State College, University Park, PA, USA; 3 Medicine and Public Health Sciences, Division of Internal Medicine, Penn State University, State College, Hershey, PA, USA; 4 Medicine, Pediatrics, and Public Health Sciences, Penn State College of Medicine, Penn State University, State College, Hershey, PA, USA; 5 Department of Public Health Sciences, Penn State Cancer Institute, Penn State University, State College, Hershey, PA, USA; 6 Human Development and Family Studies, Penn State University, State College, University Park, PA, USA; 7 Pediatrics and Public Health Sciences, Penn State College of Medicine, Penn State University, State College, Hershey, PA, USA; 8 College of Nursing, Penn State University, State College, PA, University Park, USA

The promise of community-engaged research (CEnR) to improve the health and well-being of populations is increasingly recognized by academic institutions and the programs that support their work. The National Institutes of Health’s Clinical and Translational Science Awards calls for the development of partnerships with collaborators outside of academia (e.g., patients, nonprofit organizations, governmental agencies, community-based clinicians and delivery systems, industry), “where and when appropriate [1].” Recognizing that optimal ways to involve communities in each stage of the translational process are not yet clear, the program also charged the clinical and translational research institutes (“hubs”) that received funding, to “develop a methodological framework for discovering, demonstrating and disseminating successful collaboration models [1].”

To inform its strategic planning, members of the Community-Engaged Research Core (CERC) of Penn State’s Clinical and Translational Research Institute (CTSI) reviewed the literature to identify processes and resources that promote academic institutions’ support for CEnR. The CERC identified myriad strategies for fostering CEnR; descriptions and assessments of institutions’ CEnR activities; and discussions of the promises, challenges, and ethics of CEnR, but no comprehensive model of academic institutional support for CEnR emerged.

**Figure 1:**
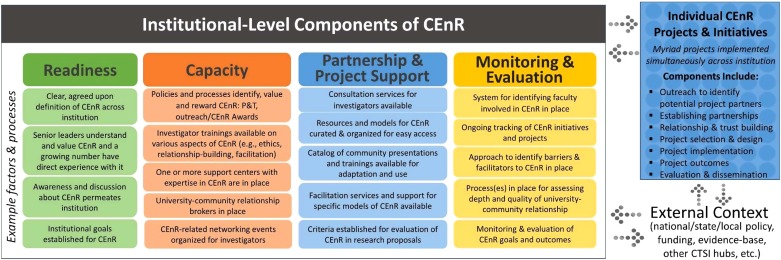
Model for academic institution support for community-engaged research (CEnR). CTSI, Clinical and Translational Research Institute; P&T, promotion and tenure.

To augment findings from the literature and to better understand the linkages between specific community engagement activities and institutional characteristics that supported or hindered them, the CERC next reviewed Web sites of CTSI hubs and conducted in-depth interviews with Penn State investigators involved in CEnR. From these efforts, the CERC developed a conceptual model (Fig. 1) to describe institutional-level components of CEnR to guide its work to promote and support CEnR at Penn State.

Four major institutional-level components of CEnR were identified in the model, each of which includes multiple factors and processes (see Fig. 1). The first 2, *readiness* and *capacity*, relate to what Minkler and Wallerstein refer to as “university context” in their conceptual model of community-based participatory research [2]. *Readiness* relates to the level of clarity about, and commitment to, CEnR within an institution. Indicators of *readiness* include senior leaders’ involvement in discussions about the practice and ethics of CEnR, and the existence of institutional goals for CEnR [3–5].

*Capacity* refers to the institution’s relationships, policies, activities, and services that provide a supportive context for developing and implementing CEnR [6], such as internal training to introduce faculty and staff to the basics of CEnR, and promotion and tenure policies that reward CEnR. *Capacity* also includes ongoing efforts that facilitate relationship building between the institution and the public (e.g., programs to assist community organizations with their research-related needs, community-academic networking opportunities designed to broker relationships).

The third component of the model, *partnership and project support*, focuses on activities, resources, and services that help to develop specific community-academic partnerships and the research projects they generate. Project support includes at-the-ready tools (e.g., training modules on core research concepts for community partners, information on developing effective CEnR proposals) for partnerships to tap and tailor, as needed [7, 8], as well as consultation and mentoring services for investigators and community partners working to develop or implement a research plan. The small box in the far, right side of the Fig. 1 represents individual CEnR projects and initiatives and identifies the major components of individual projects. The box acknowledges that individual projects may be implemented in isolation, but are helped or hindered by the institutional context in which they are situated. Importantly, individual projects can create momentum for strengthening institutional supports for CEnR by demonstrating their value, developing solutions for navigating past institutional barriers, etc.

The final institutional-level component of the model, *monitoring & evaluation*, involves the institution’s activities to understand where, with whom, how, and how much CEnR is occurring, as well as to assess the quality and outcomes of those efforts. These activities provide feedback necessary to address issues impeding CEnR (e.g., resource constraints, barriers to trust-building with community partners), and to inform planning and goal-setting [9, 10].

Finally, the model notes the influence of the institution’s external environment (e.g., the fast-growing scientific literature, public policy, meetings and information-sharing involving multiple CTSI hubs) on its values, plans, and activities.

Although future work is needed to articulate the many topics that fall within each component area, this introduction of the model may further the national conversation about CEnR by situating its component parts within a larger institutional context and may help academic institutions assess their current CEnR activities and plan for the future. Specifically, this model may advance the field of CEnR by providing institutions with a framework for cataloging measurement tools for evaluating the scope, ethics, rigor, and effectiveness of their CEnR efforts.
